# Supplementary material for: Permissive central tolerance plus defective peripheral checkpoints license pathogenic memory B cells in CASPR2-antibody encephalitis
Source: Sci Adv. 2025 Apr 16;11(16):eadr9986. doi: 10.1126/sciadv.adr9986 (PMC12002137; doi:10.1126/sciadv.adr9986)
Supplement: Supplementary file 1 — Figs. S1 to S4 Tables S1 to S5 [file sciadv.adr9986_sm.pdf]

Science Advances
AAAS

Supplementary Materials for

Permissive central tolerance plus defective peripheral checkpoints license pathogenic memory B cells in CASPR2-antibody encephalitis

Bo Sun *et al.*

Corresponding author: Sarosh R. Irani, irani.sarosh@mayo.edu

Sci. Adv. **11**, eadr9986 (2025)
DOI: 10.1126/sciadv.adr9986

This PDF file includes:

Figs. S1 to S4
Tables S1 to S5

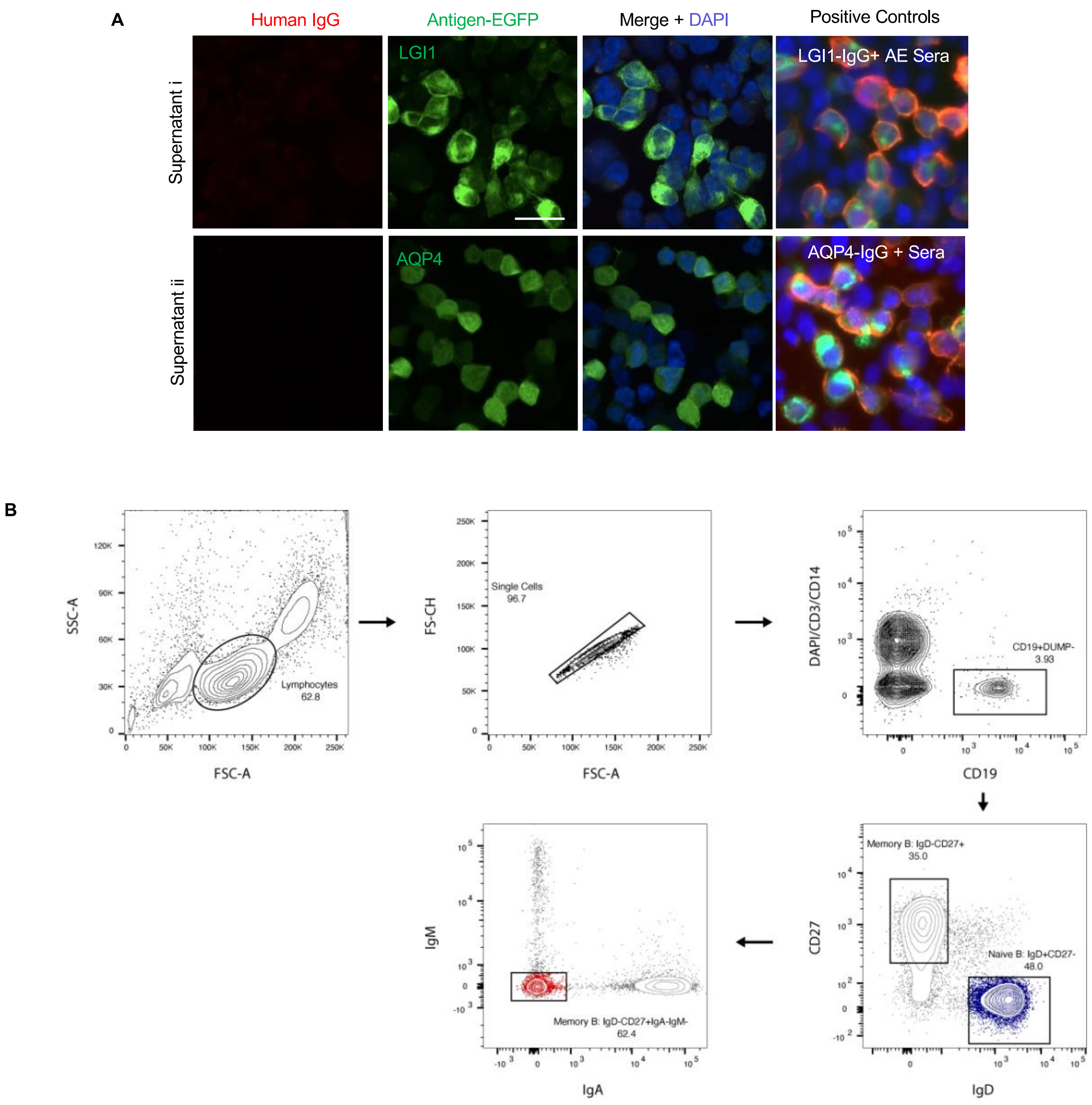

Figure S1: B cell sorting and supernatant screening

(A) Representative images demonstrating that bulk culture supernatants with CASPR2-reactivities showed no binding to other neuronal autoantigens, including LGI1 and AQP4. Positive control serum from an LGI1 antibody-positive patient and an AQP4-antibody positive patient are depicted (right). Scale bar = 1 micron. **(B)** Flow cytometry gating strategy for single cell cultures.

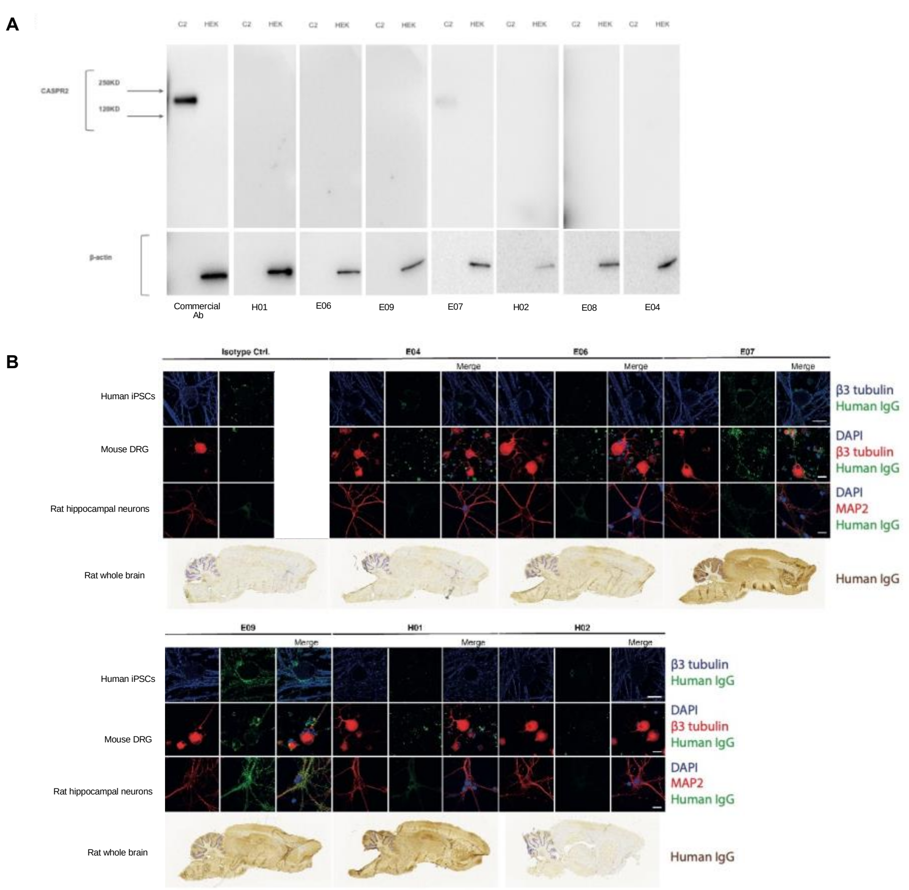

C

	CASPR2-Ab E B Cell Subset	Human iPSC Sensory Neuron	Rodent DRG	Rodent Hippocampal	Rodent Brain
B05	Naïve		-	-	-
B06	Naïve		-	-	-
B08	Naïve		-	-	-
C02	Naïve		-	-	-
C04	Naïve		-	-	-
C06	Naïve		-	-	-
C07	Naïve		-	-	-
D02	Naïve		-	-	-
D03	Naïve		-	-	-
D06	Naïve		-	-	-
F02	Naïve		-	-	-
F04	Naïve		-	-	-
F08	Naïve		-	-	-
G04	Naïve		-	-	-
G08	Naïve		-	-	-
G09	Naïve		-	-	-
E06	Memory	+	-	-	-
H02	Memory	+	-	-	-
E04	Memory	+	-	-	-
H01	Memory	+	+	-	-
E07	Memory	+	+	+	+
E08	Memory	+	+	+	+
E09	Memory	+	+	+	+

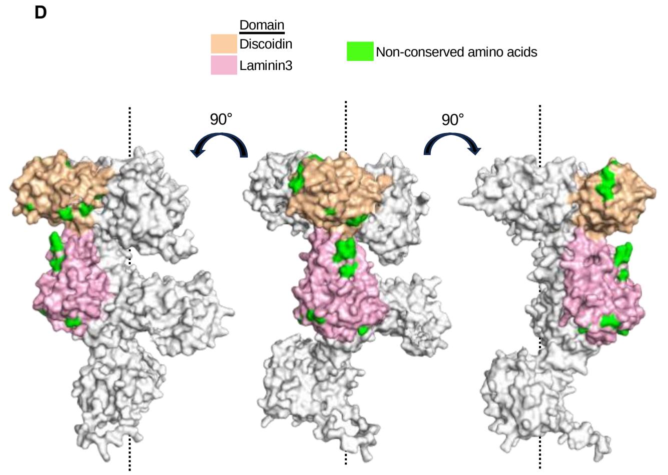

Figure S2: mAb binding to conformational epitopes

(A) Representative western blot of mAb binding to purified CASPR2 protein (C2) or HEK cell lysate (HEK). Beta-actin as protein loading control. **(B)** Remaining representative images of CASPR2 mAbs binding to live iPSC-derived human sensory neurons (first row; identified with Beta-tubulin III in blue), live mouse primary dorsal root ganglia (DRG**;** second row; Beta-tubulin III in red), live primary rat hippocampal neurons (third row; identified with microtubule associated protein 2, MAP2, red) and paraformaldehyde-fixed sagittal sections of rat whole

brains. Binding patterns are summarized in **(C). (D)** The predicted CASPR2 protein structure is rotated along the depicted axis to better visualize the entirety of the discoidin (tan) and laminin 3 (pink) domains. Surface exposed non-conserved amino acids are depicted in green.

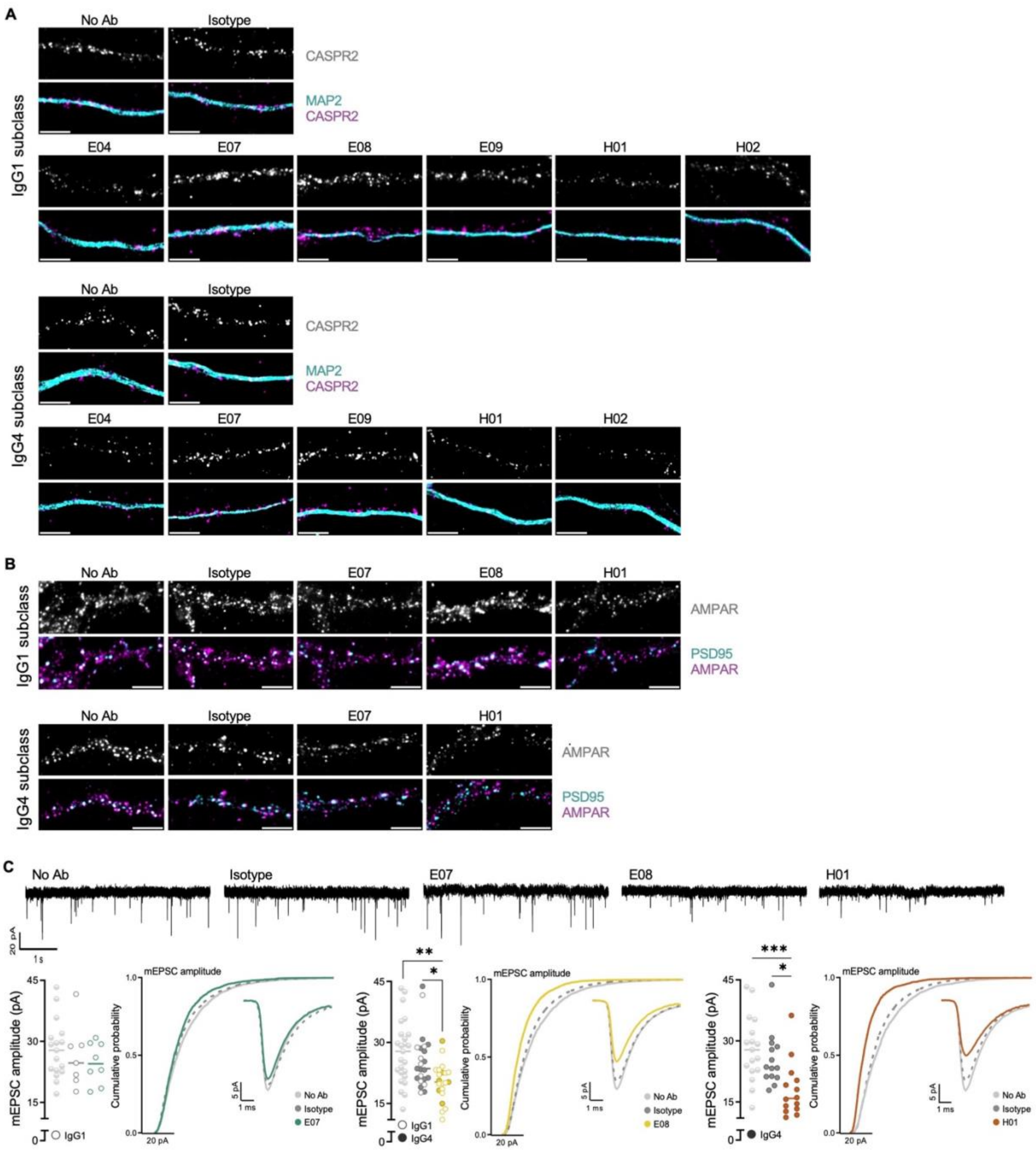

Figure S3. Diverse effects of CASPR2 mAbs on CASPR2 and AMPAR expression, and synaptic currents.

(A) Representative images of CASPR2 and MAP2 expression after 2 hour application of the various IgG1 (top) or IgG4 mAbs (bottom) quantified in Fig.4C; Scale bar = 5 µm. **(B)** Representative images of synaptic AMPAR and PSD95 expression following incubation of the E07, E08 and H01 mAbs in either IgG1 (top) or IgG4 (bottom) subclass, as quantified in Fig.4D. **(C)** Representative tracings (top) of AMPAR-mediated mEPSC recordings of pyramidal neurons following incubation of the E07, E08 and H01 mAbs in different subclasses, pooled together in

Fig.4E. (Bottom) Average amplitude quantifications, cumulative probability and single average event of cells incubated with E07 (left), E08 (middle) or H01 (right) mAbs. *=$p<0.05$, **=$p<0.01$ and ***=$p<0.001$, Kruskal-Wallis test with Dunn's multiple comparison post-hoc test.

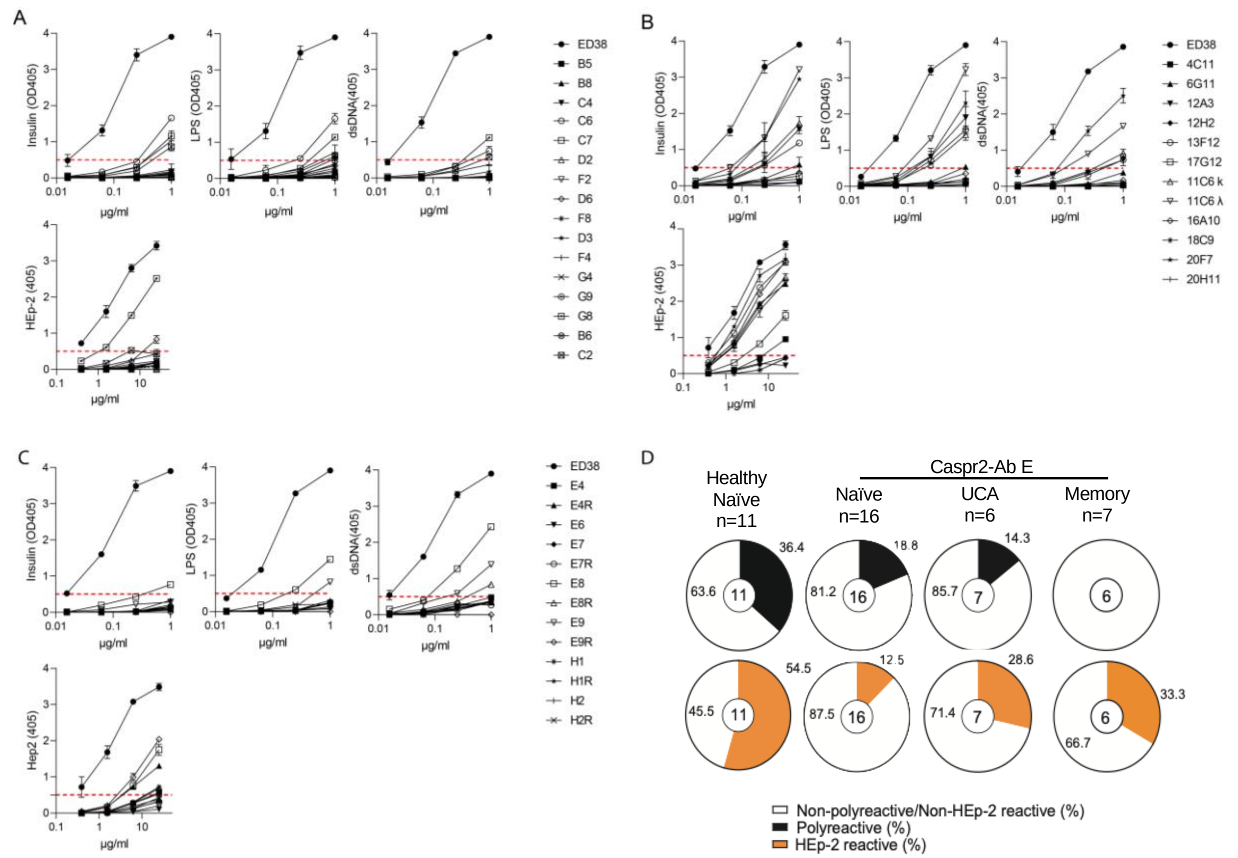

Figure S4. Insulin/LPS/dsDNA and HEp-2 cell lysate binding by CASPR2 mAbs
ELISA quantification of mAb binding to insulin/LPS/dsDNA (top) and Hep-2 cell lysate (bottom) by naïve B cell mAbs from CASPR2-Ab E patients **(A),** healthy control mAbs **(B),** and memory B cell mAbs from CASPR2-Ab E patients **(C).** Each data point represents the mean value of two independent experiments and the error bars represent SDs. Dotted horizontal red lines mark the positive reactivity cut-off at OD405 0.5. ED38, a monoclonal antibody cloned from a VpreB + L + peripheral B cell, was a positive control. **(D)** Pie charts display the overserved frequency distributions for insulin/LPS/dsDNA reactivity (black, left), or Hep-2 reactivity (right, orange). The number in the center of the pie chart represents the total number of unique mAbs tested.

Table S1: Patient demographics.

Tabular description of CASPR2-Ab E patients (labeled P1-P6), LGI1-Ab E patients (P7-P10), NMDAR-Ab E patients (P11-14) healthy controls (labeled H1-H6). M = male, F = female, Cog = cognitive impairment, Psy = psychiatric features, PNH = peripheral nerve hyperexcitability, ANS = autonomic nervous system dysfunction, BPH = benign prostatic hypertrophy, T2DM = Type 2 diabetes mellitus, HTN = hypertension, AF = atrial fibrillation, NBC = naive b cell, MBC = memory b cell, N/A = not applicable, - = not performed

ID	IgG serostatus	IgM serostatus	Sex	Age (years)	Disease duration (years)	CASPR2-Ab E Clinical Syndrome	Treatment at time of sample collection	Co-morbidities	Total wells NBC (Bulk)	Total wells MBC (Bulk)	Total wells NBC (single-cell)	Total wells MBC (single-cell)
P1	CASPR2	Negative	M	63	2	Cog, seizures	Nil	Nil	9	11	2112	2208
P2	CASPR2	Negative	M	75	7	Cog, seizures, pain	Nil	BPH, T2DM	2	6	2400	1598
P3	CASPR2	Negative	M	69	1	Cog, Psy, Pain, PNH	Nil	BPH, HTN, AF	10	1	-	-
P4	CASPR2	Negative	M	75	1	Cog, Psy, seizures	Corticosteroids	BPH, Gout, HTN	6	5	-	-
P5	CASPR2	Negative	M	71	0.75	Cog, Psy, seizures	Corticosteroids	T2DM	2	1	-	-
P6	CASPR2	Negative	M	81	7	Cog, seizures, ANS	Methotrexate	BPH, HTN, AF	2	1	-	-
P7	LGI1	Negative	M	70	N/A	N/A	N/A	LGI1-Ab E	2	1	-	-
P8	LGI1	Negative	M	53	N/A	N/A	N/A	LGI1-Ab E	4	3	-	-
P9	LGI1	Negative	F	46	N/A	N/A	N/A	LGI1-Ab E	7	3	-	-
P10	LGI1	Negative	F	52	N/A	N/A	N/A	LGI1-Ab E	16	1	-	-
P11	NMDAR	Negative	F	17	N/A	N/A	N/A	NMDAR-Ab E	13	4	-	-
P12	NMDAR	Negative	F	24	N/A	N/A	N/A	NMDAR-Ab E	10	4	-	-
P13	NMDAR	Negative	F	20	N/A	N/A	N/A	NMDAR-Ab E	7	4	-	-
P14	NMDAR	Negative	F	19	N/A	N/A	N/A	NMDAR-Ab E	23	3	-	-
H1	Negative	Negative	N/A	N/A	N/A	N/A	N/A	N/A	25	1	-	-
H2	Negative	Negative	N/A	N/A	N/A	N/A	N/A	N/A	23	4	-	-
H3	Negative	Negative	N/A	N/A	N/A	N/A	N/A	N/A	56	2	-	-
H4	Negative	Negative	N/A	N/A	N/A	N/A	N/A	N/A	30	3	-	-
H5	Negative	Negative	M	46	N/A	N/A	N/A	N/A	-	-	960	960
H6	Negative	Negative	F	33	N/A	N/A	N/A	N/A	-	-	960	960

Table S2: CASPR2 mAbs

Tabular description of recovered mAbs , with structural information obtained by IMGT High Vquest. NBC = naïve B cell, MBC = memory B cell

mAb	Donor	Subset	IGHV	IGHJ	H DNA Mutations	IG K/L V	IG K/L L	L DNA Mutations
11C6	H5	NBC	IGHV1-18	IGHJ1*01	0	IGKV3-15	IGKJ4*01	0
16A10	H5	NBC	IGHV1-46	IGHJ6*02	2	IGLV3-1	IGLJ2*01	0
18C9	H5	NBC	IGHV4-59	IGHJ4*02	0	IGLV3-21	IGLJ1*01	0
20F7	H5	NBC	IGHV3-9	IGHJ6*02	0	IGLV3-19	IGLJ2*01	2
20H11	H5	NBC	IGHV3-9	IGHJ4*02	0	IGKV1-5	IGKJ1*01	0
12A3	H6	NBC	IGHV3-43D	IGHJ4*02	0	IGLV3-21	IGLJ2*01	0
12H2	H6	NBC	IGHV3-9	IGHJ4*02	0	IGKV1-17	IGKJ4*01	0
13F12	H6	NBC	IGHV4-31	IGHJ5*02	0	IGKV1-39	IGKJ1*01	0
17G12	H6	NBC	IGHV3-9	IGHJ6*02	0	IGKV3-20	IGKJ2*01	1
4C11	H6	NBC	IGHV3-7	IGHJ4*02	0	IGLV3-25	IGLJ2*01	0
6G11	H6	NBC	IGHV3-33	IGHJ6*02	0	IGLV3-19	IGLJ2*01	0
E04	P1	MBC	IGHV3-23	IGHJ4*02	10	IGLV3-1	IGLJ2*01	13
E06	P1	MBC	IGHV2-5	IGHJ5*02	13	IGKV3-11	IGKJ2*01	15
E07	P1	MBC	IGHV3-30	IGHJ6*02	10	IGLV1-47	IGLJ1*01	13
E08	P1	MBC	IGHV3-23	IGHJ6*02	21	IGKV1-39	IGKJ2*01	15
E09	P1	MBC	IGHV4-34	IGHJ4*02	5	IGKV1-5	IGKJ2*01	4
F02	P1	NBC	IGHV3-53	IGHJ6*02	0	IGKV1-39	IGKJ3*01	0
F04	P1	NBC	IGHV4-31	IGHJ4*02	0	IGKV3-20	IGKJ1*01	0
F08	P1	NBC	IGHV3-33	IGHJ4*02	0	IGLV3-25	IGLJ3*02	6
G04	P1	NBC	IGHV1-8	IGHJ1*01	0	IGLV3-1	IGLJ1*01	4
G08	P1	NBC	IGHV4-34	IGHJ6*02	0	IGLV1-47	IGLJ1*01	2
G09	P1	NBC	IGHV3-7	IGHJ6*02	0	IGLV3-1	IGLJ1*01	4
H01	P2	MBC	IGHV3-23	IGHJ3*01	23	IGLV1-47	IGLJ1*01	18
H02	P2	MBC	IGHV3-21	IGHJ3*01	37	IGLV3-19	IGLJ3*02	29
H03	P2	MBC	IGHV3-23	IGHJ3*01	23	IGLV1-47	IGLJ1*01	18
H04	P2	MBC	IGHV3-21	IGHJ3*01	37	IGLV3-19	IGLJ3*02	29
H05	P2	MBC	IGHV3-23	IGHJ3*01	23	IGLV1-47	IGLJ1*01	18
B05	P2	NBC	IGHV4-61	IGHJ2*01	0	IGLV1-47	IGLJ3*02	2
B06	P2	NBC	IGHV3-30	IGHJ5*02	1	IGLV3-1*01	IGLJ1*01	0
B08	P2	NBC	IGHV3-33	IGHJ4*02	0	IGKV3-11	IGKJ3*01	1
C02	P2	NBC	IGHV4-61	IGHJ6*02	0	IGKV1-8	IGKJ1*01	0
C04	P2	NBC	IGHV4-31	IGHJ5*02	0	IGLV1-40	IGLJ3*02	3
C06	P2	NBC	IGHV3-21	IGHJ6*02	0	IGKV1-39	IGKJ1*01	1
C07	P2	NBC	IGHV3-48	IGHJ6*02	0	IGLV3-1	IGLJ1*01	4
D02	P2	NBC	IGHV3-7	IGHJ4*02	0	IGKV1-39	IGKJ2*01	1
D03	P2	NBC	IGHV3-23	IGHJ6*02	0	IGLV3-1	IGLJ1*01	5
D06	P2	NBC	IGHV3-9	IGHJ3*02	0	IGKV1-39	IGKJ1*01	5

Table S3: mAb binding kinetics

Table S3: Surface plasma resonance binding quantifications of memory B cell derived mAbs and their corresponding UCAs. E06-UCA heavy and light chain vector transfection did not produce mAbs in cell culture supernatant. Binding not detected = ND

Antibody	Ka ($M^{-1}s^{-1}$)	Kd (s^{-1})	K_D (M)	t 1/2 (s)
E09	3.04E+05	4.64E-05	1.53E-10	1.49E+04
E09-UCA	5.09E+04	1.70E-04	3.33E-09	4.08E+03
E08	3.70E+08	1.11E+01	3.00E-08	6.25E-02
E08-UCA	6.38E+04	8.92E-02	1.40E-06	7.77E+00
E07	3.14E+03	1.54E-04	4.90E-08	4.51E+03
E07-UCA	2.60E+03	3.99E-03	1.54E-06	1.74E+02
H02	4.79E+05	2.62E-01	5.47E-07	2.64E+00
H02-UCA	ND	ND	ND	ND
H01	1.63E+07	1.37E+01	8.37E-07	5.07E-02
H01-UCA	ND	ND	ND	ND
E04	3.21E+04	1.72E-01	5.37E-06	4.03E+00
E04-UCA	ND	ND	ND	ND
E06	1.08E+03	2.89E-01	2.67E-04	2.40E+00

Table S4: PCR primers

Primer name, target, sequence and PCR reaction from lysed B cells

Name	Chain	Primer Sequences (5' to 3')	PCR Reaction
CHG1	IgG H	GTTGTCCACCTTGGTGTTGCTGG	1
VHL1	IgG,M H	CCATGGACTGGACCTGGAG	1
VHL1 mod	IgG M H	CCATGGACTGSACCTGGAG	1
VHL2	IgG,M H	ATGGACATACTTTGCTCCAC	1
VHL2 mod	IgG M H	ATGGACATACTTTGYTCCAC	1
VHL3	IgG,M H	CCATGGAGTTKGGGCTGAGCTGG	1
VHL4	IgG,M H	ATGAAACACCTGTGGTTCTT	1
VHL4 mod	IgG M H	ATGAAACAYCTGTGGTTCTT	1
VHL5	IgG,M H	ATGGGGTCAACCGCCATCCT	1
CHm1	IgG M	GAAGCCAGCACCTGTGAGG	1
CK1	Kappa	ACACTCTCCCCTGTTGAAGCTCTT	1
VKL1	Kappa	CTCAGCTCCTGGGGCTCC	1
VKL1 mod	Kappa	CTCAGCTCCTGGGGCTYC	1
VKL2	Kappa	CTGCTCAGCTCCTGGGGC	1
VKL2 mod	Kappa	CTGCTCAGCTCYTGGGGC	1
VKL3	Kappa	GGAARCCCCAGCDCAGC	1
VKL4	Kappa	CTCTGTTGCTCTGGATCTCTG	1
VKL5	Kappa	GGGGTCCCAGGTTCACCTCCTC	1
CL1	Lambda	TGAACATTCTGTAGGGGCCAC	1
VLL1	Lambda	CCTCTCCTCCTCACCCTCCTC	1
VLL1 mod	Lambda	CCTCTCCTCCTSACCCTCCTC	1
VLL2	Lambda	CTCCTCACTCAGGGCACAG	1
VLL2 mod	Lambda	CTCCTCACTCAGGRCACAG	1
VLL3	Lambda	ATGGCCTGGAYCCCTCTCCTSCT	1
VLL3 mod	Lambda	ATGGCCTGGAYCCCTCTCCTBCT	1
VLL4a	Lambda	CTCCTCCTCTTCCCCCTCCCCCTC	1
VLL4a mod	Lambda	CTCCTCCTCCACTGCACAG	1
VLL4b	Lambda	TGGCCTGGGTCTCCTTCTACCTAC	1
VLL569	Lambda	ATGGCCTGGRCTCCTCTCCTYCTC	1
VLL710	Lambda	ATGGCCTGGRCTCCTCTCCTYCTG	1
VLL8	Lambda	ATGGCCTGGATGATGCTTCTCCTC	1
CPEC VH1/5/7	Heavy	CTTTTTCTAGTAGCAACTGCAACCGGTGTACATTCCGAGGTGCAGCTGGTGCAG	2
CPEC VH2	Heavy	CTTTTTCTAGTAGCAACTGCAACCGGTGTACATTCCCAGGTCACCTTGAAGGAG	2
CPEC VH3	Heavy	CTTTTTCTAGTAGCAACTGCAACCGGTGTACATTCTGAGGTGCAGCTGGTGGAG	2
CPEC VH4	Heavy	CTTTTTCTAGTAGCAACTGCAACCGGTGTACATTCCCAGGTGCAGCTGCAGGAG	2
CPEC VH3-23	Heavy	CTTTTTCTAGTAGCAACTGCAACCGGTGTACATTCTGAGGTGCAGCTGTTGGAG	2
CPEC VH4-34	Heavy	CTTTTTCTAGTAGCAACTGCAACCGGTGTACATTCCCAGGTGCAGCTACAGCAGTG	2
CPEC VH1–18/69 (silent	Heavy	CTTTTTCTAGTAGCAACTGCAACCGGTGTACATTCCCAGGTTCAGCTGGTGCAG	2
CPEC VH1-45/1-58	Heavy	CTTTTTCTAGTAGCAACTGCAACCGGTGTACATTCCCAGATGCAGCTGGTGCAG	2
CPEC VH1–24	Heavy	CTTTTTCTAGTAGCAACTGCAACCGGTGTACATTCCCAGGTCCAGCTGGTACAG	2
CPEC VH3–9/30/33	Heavy	CTTTTTCTAGTAGCAACTGCAACCGGTGTACATTCTGAAGTGCAGCTGGTGGAG	2
CPEC VH6–1	Heavy	CTTTTTCTAGTAGCAACTGCAACCGGTGTACATTCCCAGGTACAGCTGCAGCAG	2
CPEC VH4–39	Heavy	CTTTTTCTAGTAGCAACTGCAACCGGTGTACATTCCCAGCTGCAGCTGCAGGAG	2
CPEC VH3–33/11/30	Heavy	CTTTTTCTAGTAGCAACTGCAACCGGTGTACATTCTCAGGTGCAGCTGGTGGAG	2
CPEC JH1/2/4/5	Heavy	GATGGGCCCTTGGTCGACGCTGAGGAGACGGTGACCAG	2
CPEC JH3	Heavy	GATGGGCCCTTGGTCGACGCTGAAGAGACGGTGACCATTG	2
CPEC JH6	Heavy	GATGGGCCCTTGGTCGACGCTGAGGAGACGGTGACCGTG	2
CPEC VK1	Kappa	CTTTTTCTAGTAGCAACTGCAACCGGTGTACATTCTGACATCCAGATGACCCAGTC	2
CPEC VK1-9/1-13	Kappa	CTTTTTCTAGTAGCAACTGCAACCGGTGTACATTCAGACATCCAGTTGACCCAGTCT	2
CPEC VK1D-43/1-8	Kappa	CTTTTTCTAGTAGCAACTGCAACCGGTGTACATTGTGCCATCCGGATGACCCAGTC	2
CPEC VK2	Kappa	CTTTTTCTAGTAGCAACTGCAACCGGTGTACATGGGGATATTGTGATGACCCAGAC	2
CPEC VK2-28/2-30	Kappa	CTTTTTCTAGTAGCAACTGCAACCGGTGTACATGGGGATATTGTGATGACTCAGTC	2
CPEC VK3-11/3D-11	Kappa	CTTTTTCTAGTAGCAACTGCAACCGGTGTACATTCAGAAATTGTGTTGACACAGTC	2
CPEC VK3-15/3D-15	Kappa	CTTTTTCTAGTAGCAACTGCAACCGGTGTACATTCAGAAATAGTGATGACGCAGTC	2
CPEC VK3-20/3D-20	Kappa	CTTTTTCTAGTAGCAACTGCAACCGGTGTACATTCAGAAATTGTGTTGACGCAGTCT	2
CPEC VK4-1	Kappa	CTTTTTCTAGTAGCAACTGCAACCGGTGTACATTCGGACATCGTGATGACCCAGTC	2
CPEC JK1/2/4	Kappa	ATGGTGCAGCCACCGTACGTTTGATYTCCACCTTGGTC	2
CPEC JK2	Kappa	ATGGTGCAGCCACCGTACGTTTGATCTCCAGCTTGGTC	2
CPEC JK3	Kappa	ATGGTGCAGCCACCGTACGTTTGATATCCACTTTGGTC	2
CPEC JK5	Kappa	ATGGTGCAGCCACCGTACGTTTAATCTCCAGTCGTGTC	2
CPEC JK150/3	Kappa	ATGGTGCAGCCACCGTACGTCTGATTTCCACCTTGGTC	2
CPEC VL1	Lambda	CTTTTTCTAGTAGCAACTGCAACCGGTTCCTGGGCCCAGTCTGTGCTGACKCAG	2
CPEC VL2	Lambda	CTTTTTCTAGTAGCAACTGCAACCGGTTCCTGGGCCCAGTCTGCCCTGACTCAG	2
CPEC VL3	Lambda	CTTTTTCTAGTAGCAACTGCAACCGGTTCTGTGACCTCCTATGAGCTGACWCAG	2
CPEC VL4/5	Lambda	CTTTTTCTAGTAGCAACTGCAACCGGTTCTCTCTCSCAGCYTGTGCTGACTCA	2
CPEC VL6	Lambda	CTTTTTCTAGTAGCAACTGCAACCGGTTCTTGGGCCAATTTTATGCTGACTCAG	2
CPEC VL7/8	Lambda	CTTTTTCTAGTAGCAACTGCAACCGGTTCCAATTCYCAGRCTGTGGTGACYCAG	2
CPEC CL reverse	Lambda	GGCTTGAAGCTCCTCACTCGAGGGYGGGAACAGAGTG	2
3'CL Reverse	Lambda	CACCAGTGTGGCCTTGTTGGCTTG	2
panVK Forw.	Kappa	ATGACCCAGWCTCCABYCWCCCTG	2
3'CK494-516 R.	Kappa	GTGCTGTCCTTGCTGTCCTGCT	2
3' CgCH Reverse Tiller	Heavy	GGAAGGTGTGCACGCCGCTGGTC	2

Table S5: Commercial antibodies

Target	Clone or laboratory number; Manufacturer	Assays	Concentration or Dilution
CD3	UCHT1; BioLegend	FACS	1:80
CD14	HCD14; BioLegend	FACS	1:80
CD19	SJ25C1; BD Biosciences	FACS	1:32
CD27	O323; BioLegend	FACS	1:16
IgD	IA6-2; BD Biosciences	FACS	1:40
CD38	HB7; BD Biosciences	FACS	1:40
DAPI	564907; BD Pharmingen	FACS; Cell based assays	FACS: 0.5 ug/ml, CBA: 1:5000
Human IgG	A11014; Thermofisher	Cell based assays	1:750
Human IgM	A21216; Thermofisher	Cell based assays	1:500
Human IgG	SAB3701279; Sigma	Brain sections	1:500
Caspr2	Ab33994; Abcam	Cortical rat neurons	1:200
MAP2	Ab5392; Abcam	Cortical rat neurons	1:5000
PSD-95	3450; Cell Signalling Technologies	Cortical rat neurons	1:750
pan-GluA	182411; Synaptic Systems	Cortical rat neurons	1:100
Chicken IgY	103-155-155; Jackson ImmunoResearch	Cortical rat neurons	1:200
Mouse IgG	A11001; Thermofisher	Cortical rat neurons	1:500
Rabbit IgG	A11036; Thermofisher	Cortical rat neurons	1:500
Human IgG	A-11013ThermoFisher	Dorsal root ganglion rat	5 µg/mL
Beta tubulin III	Ab179511; Abcam	Dorsal root ganglion rat	2 µg/mL
Human IgG	31125; ThermoFisher	Hippocampal rat neurons	2 and 3 µg/mL
Goat IgG	A11055; ThermoFisher	Hippocampal rat neurons	1:750
MAP2	ab5392; Abcam	Hippocampal rat neurons	(1:3000)
Chicken IgY	A11041; ThermoFisher	Hippocampal rat neurons	3 µg/mL
Human IgG (Fc)	SA5-10134; ThermoFisher	Sensory-neuron IPSCs	1:500
Goat IgG	A11055, ThermoFisher	Sensory-neuron IPSCs	1:500
Beta tubulin III	T2200; Sigma	Sensory-neuron IPSCs	1:2000
Rabbit IgG	P-10994; ThermoFisher	Sensory-neuron IPSCs	1:1000
